# Predicting the Temperature Evolution during Nanomilling of Drug Suspensions via a Semi-Theoretical Lumped-Parameter Model

**DOI:** 10.3390/pharmaceutics14122840

**Published:** 2022-12-18

**Authors:** Gulenay Guner, Dogacan Yilmaz, Helen F. Yao, Donald J. Clancy, Ecevit Bilgili

**Affiliations:** 1 Otto H. York Department of Chemical and Materials Engineering, New Jersey Institute of Technology, Newark, NJ 07102, USA; 2 Department of Mechanical and Industrial Engineering, New Jersey Institute of Technology, Newark, NJ 07114, USA; 3 GlaxoSmithKline, Drug Product Development, Collegeville, PA 19426, USA

**Keywords:** nanomilling, wet stirred media mill, drug nanoparticles, lumped parameter model, process modeling, heat generation, cooling

## Abstract

Although temperature can significantly affect the stability and degradation of drug nanosuspensions, temperature evolution during the production of drug nanoparticles via wet stirred media milling, also known as nanomilling, has not been studied extensively. This study aims to establish both descriptive and predictive capabilities of a semi-theoretical lumped parameter model (LPM) for temperature evolution. In the experiments, the mill was operated at various stirrer speeds, bead loadings, and bead sizes, while the temperature evolution at the mill outlet was recorded. The LPM was formulated and fitted to the experimental temperature profiles in the training runs, and its parameters, i.e., the apparent heat generation rate *Q*_gen_ and the apparent overall heat transfer coefficient times surface area *UA*, were estimated. For the test runs, these parameters were predicted as a function of the process parameters via a power law (PL) model and machine learning (ML) model. The LPM augmented with the PL and ML models was used to predict the temperature evolution in the test runs. The LPM predictions were also compared with those of an enthalpy balance model (EBM) developed recently. The LPM had a fitting capability with a root-mean-squared error (RMSE) lower than 0.9 °C, and a prediction capability, when augmented with the PL and ML models, with an RMSE lower than 4.1 and 2.1 °C, respectively. Overall, the LPM augmented with the PL model had both good descriptive and predictive capability, whereas the one with the ML model had a comparable predictive capability. Despite being simple, with two parameters and obviating the need for sophisticated numerical techniques for its solution, the semi-theoretical LPM generally predicts the temperature evolution similarly or slightly better than the EBM. Hence, this study has provided a validated, simple model for pharmaceutical engineers to simulate the temperature evolution during the nanomilling process, which will help to set proper process controls for thermally labile drugs.

## 1. Introduction

Low water solubility is a characteristic of most of the newly discovered active pharmaceutical ingredients (APIs) [1,2,3]. The APIs with low solubility have been regarded as highly risky development candidates [4]; their development into a marketed product has been more challenging, regardless of how desired their pharmacological properties are [5]. Nevertheless, either due to high-throughput screening technologies [6,7] or the need for high lipophilicity and molecular weight for the treatment of bio-complex diseases [8], the low solubility issue remains a challenge [9]. Therefore, there is increasing motivation to study solubility and dissolution enhancement methods by pharmaceutical companies and scientists [10]. Some of the popular methods entail formulation of amorphous solid dispersions [11,12], co-crystals [13,14], and nanoparticles [15,16]. The focus of this paper is drug nanoparticle suspensions (nanosuspensions), and their most preferred production method, i.e., wet stirred media milling (WSMM) [15].

Whereas WSMM results in the breakage of drug particles caused by bead collisions, the stability of the nanoparticles emerges as a big challenge, which has been the most studied aspect of the process [15,16]. On the other hand, process-related issues are not as widely considered in the literature [17]. Among process-related problems, there are only a few studies related to scale-up [18,19,20] and media wear/contamination [21,22,23], whereas breakage kinetics and its relation to various process conditions have been widely studied in the WSMM literature [18,24,25,26,27,28,29,30]. Recently, a well-known but overlooked issue of the WSMM process, heat generation caused by viscous losses and collisions of beads with themselves and the mill wall [31,32], has been addressed for the first time in the pharmaceutical nanotechnology literature [33,34]. Temperature rise during milling is a critical issue since it can facilitate Ostwald ripening and growth [35,36,37], amorphization [38,39], or cause precipitation of stabilizers when gelation temperature is exceeded [40,41]. Despite the criticality of this issue, it is surprising that except for two studies [33,34], the majority of the WSMM studies did not delve into heat generation–transfer aspects of the process. It should be noted that process conditions such as stirrer speed and bead loading have a significant impact on the timewise evolution of temperature and the maximum temperatures attained during the WSMM [34].

Modeling is important for gaining a process understanding of the WSMM. The models for the WSMM could be categorized as mechanistic models, statistically based models, and semi-theoretical models [17]. For the modeling of breakage kinetics, mechanistic models such as population balance model [42,43] and discrete element method [44,45] have been used as well as statistically based models such as regression fits via the 1st order breakage model [46,47] and the nth order breakage model [30,48]. In the last decade, our group elucidated the impact of the process parameters via a mechanistic, microhydrodynamic model [16,29,30]. An example of a hybrid semi-theoretical model could be the prediction of breakage rate constant in the nth order model, based on microhydrodynamic parameters [49,50]. Semi-theoretical models can be advantageous as they provide practical information about the process, without having high computational cost. Furthermore, compared to purely statistically based, or empirical models, they can do more accurate predictions thanks to their theoretical foundation [49,50]. In addition to these models, machine learning (ML) algorithms have found significant use in various fields including pharmaceuticals (e.g., [51,52,53,54]).

As heat generation analysis during WSMM is a new topic, there has been only one modeling attempt for examining the temperature rise during WSMM [33] by using an enthalpy balance model (EBM), which was performed by our group. The EBM necessitates the simultaneous solution of five ordinary differential equations (ODEs) along with a sophisticated optimizer for parameter estimation. The EBM considers all salient physical features of the process, such as the recirculating suspension, the configuration of the mill, and the jacketed cooling of the mill and the holding tank, while requiring all physical–thermal properties of the suspension and mill–bead materials of construction as input to the model [33]. It is fair to state that the EBM is not a simple model and requires some notable time and effort, justifying the development of simpler models.

In this study, we aim to develop a simple semi-theoretical model to simulate and predict the temperature evolution during the WSMM, which can be easily adopted by pharmaceutical scientists and engineers. To this end, we formulated a lumped-parameter model (LPM) and compared its performance to the previously developed EBM. The experiments consisted of 32 different combinations of stirrer speed, bead loading, and bead size of which 27 were used for model training and 5 were reserved for model testing. The LPM parameters, i.e., the apparent heat generation rate *Q*_gen_ and the apparent overall heat transfer coefficient times surface area *UA*, were obtained by direct fitting of the LPM to the experimentally measured temperature profiles via SigmaPlot. The parameters estimated for the 27 process runs enabled us to train the power law (PL) and the machine learning (ML) model. By using the trained models, we predicted *Q*_gen_ and *UA* of the five test runs. Minitab was used for the PL predictions, whereas Google Colab was used for the ML predictions. By inserting the predicted *Q*_gen_ and *UA* values in the LPM, the temperature profiles of the test runs were simulated. The advantages and disadvantages of the LPM and the EBM as well as the limitations of the LPM were discussed. Not only will this study reveal the fitting capability of the LPM as compared with the EBM, but it will also enable us to assess their comparative predictive capabilities and usefulness for process development and understanding. Overall, this study provides pharmaceutical engineers with a validated, simple model (LPM), which simulates the temperature evolution during the production of drug nanosuspensions and predicts the impact of process parameters, thereby eventually helping engineers to control and optimize the process.

## 2. Materials and Methods

### 2.1. Materials

A BCS Class II API, fenofibrate (FNB) was used as a model poorly water-soluble drug, which was purchased from Jai Radhe Sales (BP grade, Ahmedabad, India). FNB is a lipophilic compound with a molecular weight of 360 g/mol and an aqueous solubility of 0.8 mg/L at room temperature [55]. Hydroxypropyl cellulose (HPC) was used for stabilizing the drug suspension as a non-ionic polymer, which was generously donated by Nisso America Inc. (L grade, New York, NY, USA). Its L grade has a molecular weight of 140,000 g/mol [56] and is readily water-soluble [57]. HPC is known to adsorb onto FNB particles, thereby imparting steric stability [58]. Moreover, an anionic surfactant, sodium dodecyl sulfate (SDS), was used for wettability enhancement, and it was purchased from GFS chemicals (ACS grade, Columbus, OH, USA). SDS has a molecular weight of 288 g/mol and a critical micelle concentration of 8 mM [55].

### 2.2. Methods

#### 2.2.1. Wet Stirred Media Milling

The suspension formulation was the same for all runs where the *w/v*% of the ingredients was 10% FNB, 8% HPC-L, and 0.05% SDS with respect to 200 mL of deionized water. Based on our prior studies, this formulation is known to be physically stable during milling and storage [30,50]. A pre-suspension was prepared by adding the powders to deionized water gradually under constant mixing with a shear stirrer (Cat#. 14-503, Fisher Scientific, Pittsburgh, PA, USA) operating at 300 rpm for 2 h. The theoretical batch size was fixed for all processing runs: 236 g. Pre-suspensions were stored at 8 °C overnight prior to milling to let it settle and get rid of the foaming that occurred during shear mixing. On the milling days, suspensions were stirred on a magnetic stirrer until they equilibrated close to room temperature.

A Microcer wet-stirred media mill (Netzsch Fine Particle Size Technology, LLC, Exton, PA, USA) was used for milling the pre-suspensions. It has an 80 mL chamber volume lined with zirconia, a zirconia shaft, and stainless-steel screens whose openings are of half the size of the yttrium stabilized zirconia beads (YSZ, Saint Gobain ZirPro, Malvern, PA, USA). A Cole-Palmer peristaltic pump (Master Flex, Vernon Hills, IL, USA) recirculated the suspension between the holding tank and mill chamber at a 126 mL/min flow rate, which was kept the same for all processing runs. The milling conditions are shown in Table 1, where 27 experiments were used as training runs and 5 additional runs were used for testing the model prediction capability. Stirrer speed, bead loading, and bead size were varied at 3 levels for the training runs. The low–high values of the stirrer speed and the bead loading were selected based on our prior wet media milling studies using FNB [36,59], the limitations of our milling equipment, and the objective of preparing drug nanosuspensions with a median particle size below 0.5 µm within 60 min. A stirrer speed lower than 2000 rpm and/or a bead loading lower than 0.4 could result in extremely low breakage rates, and in turn coarser particles with a median size greater than 0.5 µm. Hence, they were excluded. The design limit of the equipment (4200 rpm) dictates 4000 rpm as the high value with a safety margin. The bead loading above 0.60 could cause significant pressure build-up and mill shut-down, and it is also constrained by the maximum packing limit of the beads (~0.63 for the randomly packed monodispersed spherical beads). The bead size range of 200–800 µm covers the range of the most widely used bead sizes in WSMM (refer to [60,61] and the references cited therein). Whereas the use of beads smaller than 200 µm can be advantageous for the production of sub100 nm particles [60], bead sizes of 50 and 100 µm in size have not been widely used in pharmaceutical manufacturing because of practical clogging issues [62] as well as dust-handling issues.

Despite the use of a chiller with an initial temperature of 6.1 °C, the temperature rise during the process was inevitable due to heat generation as the drug suspension with the beads was stirred. Milling was started when the chiller temperature reached 6.1 °C, and the mill outlet temperature was equal to or below 18 °C. Even though keeping the initial temperatures for both the chiller and the mill outlet the same would be a better approach, only the initial chiller temperature could be kept the same; the initial temperature at the mill outlet varied in a narrow range (13–18 °C) because of variation in the ambient temperature, the pre-suspension temperature, and operator practice. During the experiments, the mill outlet temperature was recorded every minute (Runs 1–17) or every 30 s (Runs 18–27). The effective milling time was the same for all runs (60 min), whereas the operating time was variable (60–380 min) because of intermitting milling to prevent temperature exceeding the gelation temperature of the polymer (45 °C) [57]. In an intermittent milling cycle, the mill was shut down while cooling continued, and whenever the mill outlet temperature reached 18 °C, the milling continued. Note that we considered only the first milling cycle in the simulations. The average power consumption *P* was calculated by dividing the total energy consumption read in the mill panel by the effective milling time.

#### 2.2.2. Formulation of the Lumped-Parameter Model (LPM)

During the milling of drug suspensions, heat is generated because of the conversion of mechanical energy input by the stirrer of the mill. The heat generated is removed by a coolant passing through the jacket of the milling chamber. Ignoring the enthalpic effects associated with the suspension recirculation between the holding tank and the milling chamber, we can come up with a simple, low-fidelity model that retains the essential elements of the heat generation–transfer. The difference between the heat generation rate and heat removal rate will cause the internal energy build-up in the mill as milling continues and temperature in the mill rises. Applying the lumped capacity method [63], within the context of a transient enthalpy balance on the mill chamber, we derived the following semi-theoretical lumped-parameter model:

(1) $$mC_{p}\frac{dT}{dt}=Q_{\mathrm{gen}}-UA\left(T-T_{\mathrm{ch}}\right)$$

where *t* is milling time, *m* is the mass in the mill chamber, *C*_p_ is the specific heat capacity, *T* is the temperature at the mill outlet, *Q*_gen_ is the apparent heat generation rate during milling, *UA* is the apparent overall heat transfer coefficient times surface area, and *T*_ch_ is the chiller temperature. Strictly speaking, Equation (1) represents a transient enthalpy balance for a perfectly mixed batch process. The perfect mixing implies that the mill outlet temperature is equal to the temperature of the suspension in the mill chamber. The well-mixedness in the milling chamber has been established as a valid approximation to the residence time distribution in small mills (small length-to-diameter ratio) [64]. Hence, for a recirculation mill operating with a fixed batch size and recirculation rate, *Q*_gen_ and *UA* may only represent the heat generation rate and overall heat transfer coefficient times surface area, in some approximate, apparent, and statistical manner because they are obtained by fitting to experimental data directly. Although *UA* can be estimated based on heat transfer correlations for the internal and external convective heat transfer coefficients [33], such correlations are approximate, and none exists for the specific stirrer–mill chamber geometry. We also assumed time-invariant, constant *Q*_gen_, *C*_p_, *UA*, and *T*_ch_ (6.1 °C).

Upon separating the variables in Equation (1), integrating both sides, and imposing the initial condition, i.e., $t=0,\;T=T_{0}$, the following equation for the time-wise evolution of mill outlet temperature (shortly temperature hereafter) was obtained:

(2) $$T=T\left(t\right)=\left(T_{\mathrm{ch}}+\frac{Q_{\mathrm{gen}}}{UA}\right)+\left(T_{0}-T_{\mathrm{ch}}-\frac{Q_{\mathrm{gen}}}{UA}\right)\exp\left(-\frac{UA}{mC_{p}}t\right)$$

Here, *m* and *C*_p_ were determined considering the materials in the mill chamber: the beads (zirconia, *C*_p_ = 0.46 J/g °C [65]), the suspension (10% FNB with respect to water, *C*_p_ = 3.93 J/g °C), and the stirrer element (zirconia, *C*_p_ = 0.46 J/g °C). Whereas the stirrer element mass was constant, the bead and suspension mass varied when bead loading was changed in various runs (refer to Table 1). The *C*_p_ was calculated as the weighted average of the *C*_p_ of individual materials and the *mC*_p_ was found to be 465.6, 455.2, 444.8, 460.4, 450.0, and 470.9 J/°C for 0.4, 0.5, 0.6, 0.45, 0.55, and 0.35 bead loadings, respectively.

#### 2.2.3. Fits by the LPM and Predictions by the LPM Augmented with the PL and ML Models

By fitting Equation (2) to the experimental *T* vs. *t* data in SigmaPlot 12, *Q*_gen_ and *UA* were estimated. Then, these parameters were mathematically expressed as a function of the process parameters for the 27 training runs and predicted as a function of the process parameters for the 5 test runs using a power law (PL) model and a machine learning (ML) model. Minitab was used for the PL predictions, whereas Google Colab was used for the ML predictions. Among the several applied machine learning approaches using Google Colab, as shown in Table S1 of Supplementary Materials, k-nearest neighborhood (KNN) [66] with *k* = 5 was selected because of its low mean squared error (MSE) and mean absolute error (MAE) compared to other methods for the test runs. Therefore, ML refers to KNN (k = 5) for the rest of this study.

## 3. Results and Discussion

### 3.1. Properties of the Milled Suspensions and Particles

As the scope of this study is the simulation of temperature rise during WSMM via a lumped-parameter model, readers are referred to previous investigations for full characterization of particle sizes, viscosity, crystallinity, and morphology of the particles after milling [33,34]. Here, it suffices to summarize the key findings. All runs yielded nanoparticles upon 60 min milling, where the median particle sizes varied between 149–400 nm (refer to Table S2 of Supplementary Materials). The HPC–SDS combination successfully stabilized the drug nanoparticles by mitigating their aggregation during milling and storage. The nanoparticles were visible in SEM, confirming the laser diffraction results. XRD results of the nanoparticles showed the characteristic peaks of as-received FNB, indicating the crystal structure of the FNB was largely preserved during the milling.

### 3.2. Fitted LPM Parameters and the Origin of Temperature Rise during the Milling

**Figure 1:**
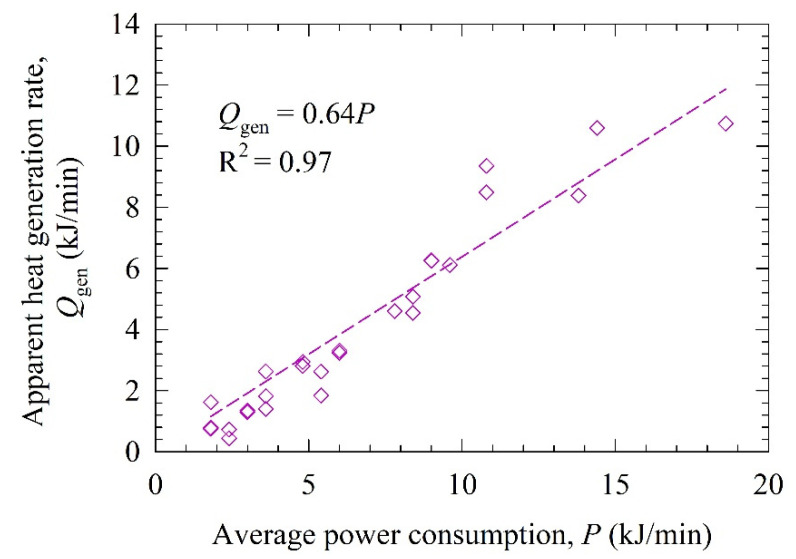
Correlation between apparent heat generation rate *Q*_gen_ and power consumption *P*.

The data on the timewise evolution of the mill outlet temperature was fitted by the LPM, as represented by Equation (2) for each training run (Runs 1–27). The fitted parameters are presented in Table 2 along with the root-mean-squared error (RMSE). The RMSE values ranged between 0.15–0.90 °C. Such low RMSE values suggest that the LPM has excellent fitting or descriptive capability of the temperature profiles despite having only two parameters. Figure 1 demonstrates that the apparent heat generation rate *Q*_gen_ is linearly and strongly correlated with the average mechanical power consumption *P* (R^2^ = 0.97). The value of the constant slope of the linear correlation in Figure 1 indicates that about 64% of the power consumption (rate of shaft work) dissipates as heat. This is not surprising at all: only a small fraction of the mechanical energy spent on mixing the suspension–bead mixture is used to deform the particles [31]. Most is converted into heat through dissipative processes such as viscous losses, inelastic bead–bead and bead–wall collisions, etc. [67]. Some of the shaft work is also spent on generating new particle surfaces (surface energy), sound, and the elastic parts of bead–bead and bead–wall collisions [33].

**Figure 2:**
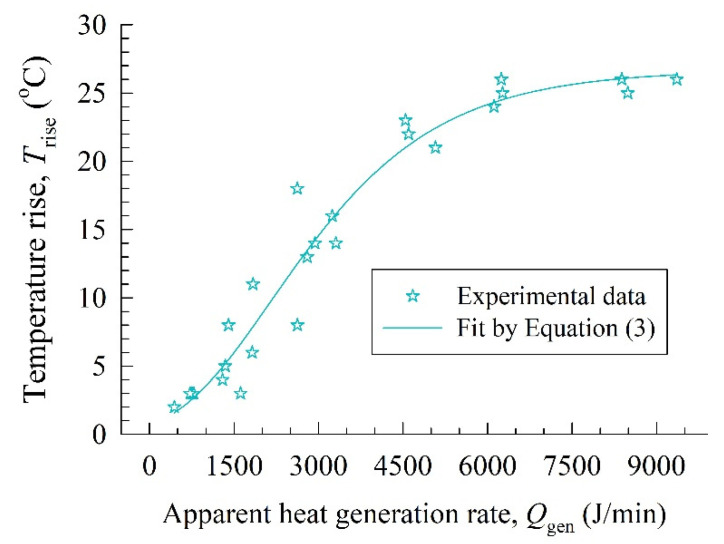
Temperature rise at 6 min as a function of the apparent heat generation rate (Runs 1–25).

Before we delve into the experimental temperature profiles and their fitting by the LPM, let us quickly assess how the temperature rise *T*_rise_ in the mill at 6 min was affected by the apparent heat generation rate *Q*_gen_ parameter of the LPM. Based on Equation (1), we expect that *Q*_gen_ is the driving force for the temperature rise, which was illustrated in Figure 2. Overall, the temperature rise was more pronounced for higher *Q*_gen_ or higher *P*, in view of Figure 1. The Gompertz growth function in Equation (3) fitted the temperature rise well (R^2^ = 0.95). As *Q*_gen_ approaches zero, it predicts a negligibly small temperature rise (~0.7 °C).

(3) $$T_{\mathrm{rise}}=26.67\exp[-\exp\left(1.30-6.02\times 10^{-4}Q_{\mathrm{gen}}\right)]$$

It is worth mentioning the caveat that the LPM is too simplistic with a multitude of assumptions; therefore, the *Q*_gen_ values do not reflect the actual heat generation rate; by and large, *Q*_gen_ is a fitting parameter affected by the accuracy of the experimental measurements and the assumptions made in the model development. On the other hand, Figure 1 and Figure 2 and the correlations therein strongly associate *Q*_gen_ with the underlying physics of the conversion of shaft work (power consumption) into heat and ensuing temperature rise. The upshot of these findings is that the LPM differs from a purely empirical model. The latter would fit temperature evolution as a function of time with parameters that have no connection to the physics of the heat generation–transfer phenomena.

### 3.3. LPM-Fitted Temperature Profiles and LPM–PL/LPM-ML Predictions in the Training Runs

**Figure 3:**
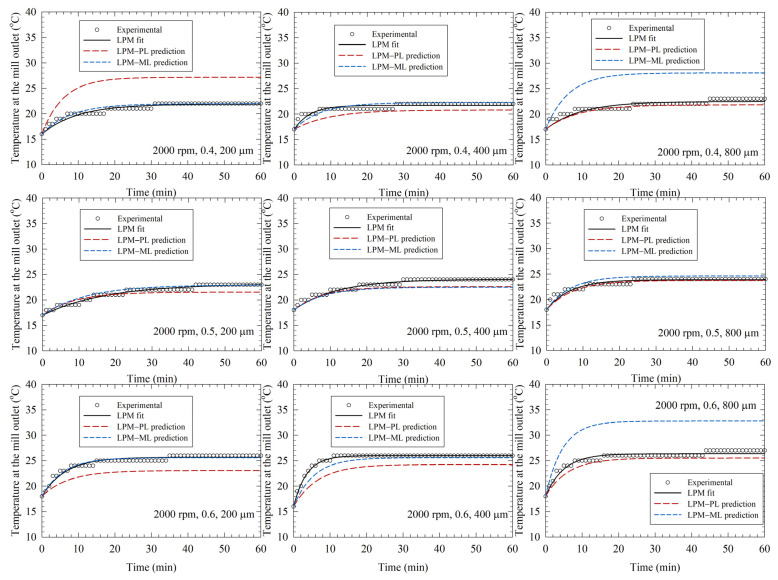
Experimental temperature profiles, direct fits by the lumped parameter model (LPM), and predictions by the LPM coupled with a power law (PL) model and a machine learning (ML) model. (**Left**)-to-(**right**): increasing bead size, (**top**)-to-(**bottom**): increasing bead loading, stirrer speed: 2000 rpm.

**Figure 4:**
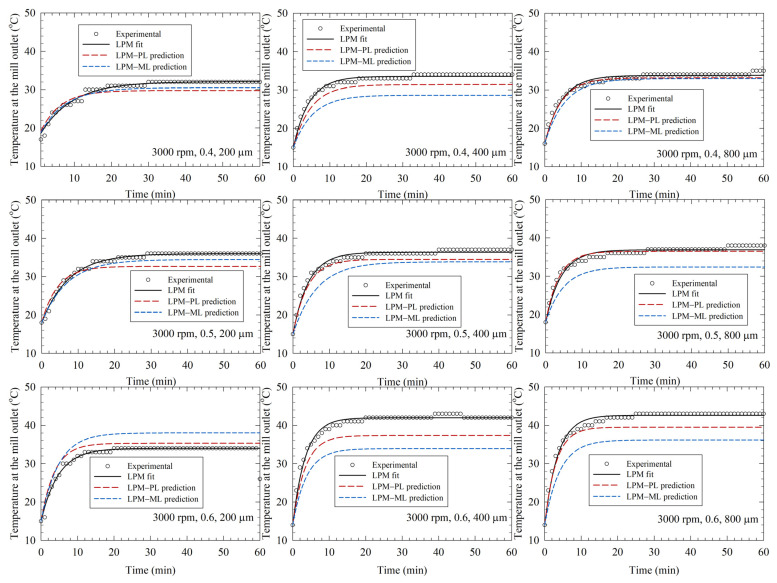
Experimental temperature profiles, direct fits by the lumped parameter model (LPM), and predictions by the LPM coupled with a power law (PL) model and a machine learning (ML) model. (**Left**)-to-(**right**): increasing bead size, (**top**)-to-(**bottom**): increasing bead loading, stirrer speed: 3000 rpm.

**Figure 5:**
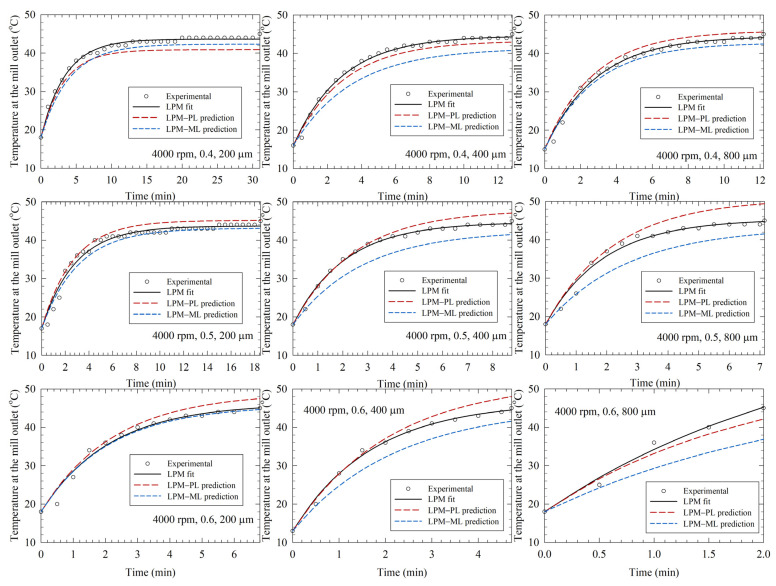
Experimental temperature profiles, direct fits by the lumped parameter model (LPM), and predictions by the LPM coupled with a power law (PL) model and a machine learning (ML) model. (**Left**)-to-(**right**): increasing bead size, (**top**)-to-(**bottom**): increasing bead loading, stirrer speed: 4000 rpm.

Figure 3, Figure 4 and Figure 5 depict the experimental time-wise evolution of the temperature profiles at various bead loadings and sizes for stirrer speeds of 2000, 3000, and 4000 rpm, respectively, and their direct fitting by the LPM. In agreement with the low RMSE values (Table 2), the fitted profiles visually corroborate that the LPM has excellent fitting capability despite its simplicity. A cursory look at the experimental temperature profiles suggests that the mill outlet temperature rose during the milling due to the conversion of the shaft work into heat and ensuing heat generation. Although the temperature rise was monotonic, the temperature attained a steady-state value for 2000 and 3000 rpm runs. The heat generation rate was so high at 4000 rpm that the mill was shut down earlier than 60 min and many intermittent milling cycles were conducted. In all profiles, the slope of the temperature profile decreased during the milling. Guner et al. [34] attributed the decreasing rate of temperature rise to a decrease in the instantaneous power consumption during the milling, which originates from the reduction of viscosity at the higher temperatures and particle size reduction during the milling [68,69]. Figure 3, Figure 4 and Figure 5 also imply that a higher stirrer speed led to higher heat generation rate; stirrer speed is the dominant process parameter, whereas the impact of the bead size is the weakest.

To quantify the impact of the process parameters, motivated by our earlier work [33], a power-law (PL) model and machine learning (ML) model were trained using the directly fitted values of *Q*_gen_ and *UA* in Runs 1–27, which were conducted at various stirrer speeds *ω*, bead loadings *c*, and the bead sizes *D*_b_. For the PL model training, Minitab was used, and Equations (4) and (5) were obtained via fitting.

(4) $$Q_{\mathrm{gen}}=6.56\times 10^{-8}\omega^{3.02}c^{1.29}D_{b}^{0.22}$$

(5) $$UA=1.32\times 10^{-4}\omega^{1.68}c^{0.77}D_{b}^{0.12}$$

Equations (4) and (5) signify through their exponents that the stirrer speed and the bead loading had the most significant impact on *Q*_gen_ and *UA*, whereas the bead size impact was much weaker. The relative impact of these parameters can be rank-ordered as follows: stirrer speed > bead loading >> bead size. It is well-known that an increase in the stirrer speed and bead loading increases the power consumption in a wet stirred media mill [31,70], which in turn leads to higher *Q*_gen_. Similarly, it is well-known that an increase in stirrer speed and bead loading also leads to an increase in the internal convective heat transfer coefficient in the mill chamber, which could lead to a higher *UA* [71,72].

By augmenting the PL model, Equations (4) and (5), and the ML model (KNN) with the LPM, we predicted the temperature profiles and compared them with the experimental profiles as well as the profiles generated by direct fitting with the LPM alone (see Figure 3, Figure 4 and Figure 5). The associated mean squared error (MSE) and mean absolute error (MAE) are reported in Table S1 of Supplementary Materials. The predictions by the LPM–PL and the LPM–ML deviated from the experimental data more than the direct fits by the LPM alone, which is intuitively expected. These deviations can be reduced by increasing the number of training runs, i.e., additional experiments at other stirred speeds and bead loadings. Both the LPM–PL and the LPM–ML predicted the profiles very well in many runs (e.g., Runs 2, 12, and 21). The LPM–PL predictions were generally closer to the experimental profiles than the LPM–ML predictions, with a few notable exceptions for each stirrer speed. The maximum deviation between the LPM–PL prediction and the experimental data ranged from 1.5 to 6.5 °C, with a mean and standard deviation of 3.1 ± 1.3 °C. Overall, the LPM successfully captured some salient qualitative patterns of the temperature profiles: (i) the monotonic temperature increase with a decreasing rate, (ii) attainment or approach to a steady-state temperature, (iii) the drastic decrease of time to reach 45 °C at 4000 rpm, and (iv) the relative impact of the process parameters.

### 3.4. Comparative Analysis of LPM and EBM Fits and Their Predictions for the Test Runs

**Figure 6:**
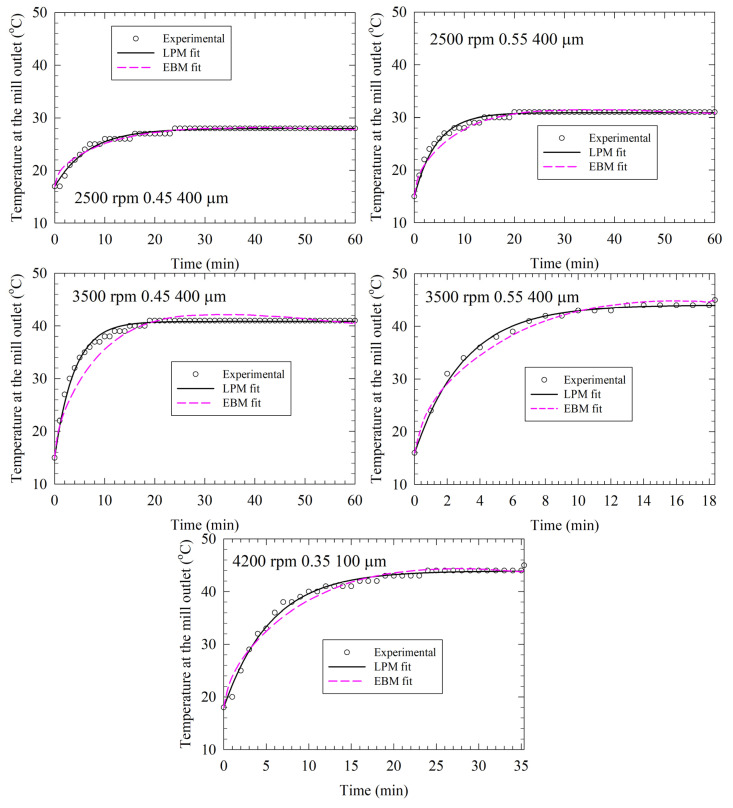
Direct fitting of the experimental temperature profiles via the lumped parameter model (LPM) and the enthalpy balance model (EBM) for the test runs (Runs 28–32). The EBM fits were taken from Guner et al. (2022) [33].

We reserved Runs 28–32 data for testing the simple LPM in comparison to the more elaborate EBM. We first fitted the LPM and the EBM to the experimental temperature profiles directly, estimated *Q*_gen_ and *UA* (see the RMSE in Table 3), and illustrated the fitted profiles in Figure 6. The EBM data were retrieved from Guner et al. [33] for comparison. Figure 6 and Table 3 data suggest that the LPM fitted the experimental temperature profiles slightly better than the EBM. The average ± standard deviation of the RMSEs are 0.50 ± 0.12 °C for the LPM and 0.96 ± 0.43 °C for the EBM. Moreover, LPM has a lower deviation from the experimental data and is more consistent, according to the lower standard deviation.

**Figure 7:**
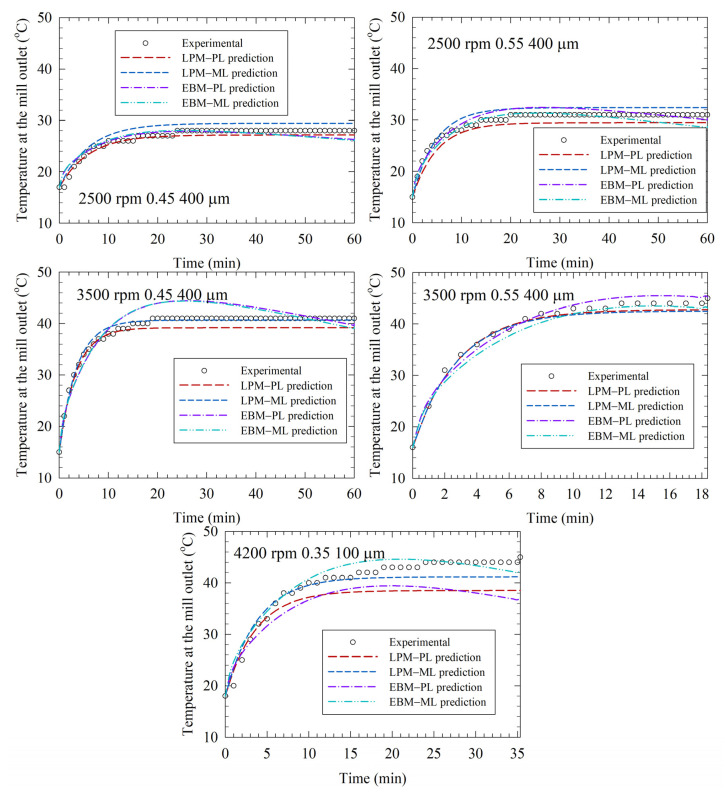
Predictions by power law (PL) and machine learning (ML) models coupled with the lumped parameter model (LPM) and the enthalpy balance model (EBM) for the test runs (Runs 28–32). The EBM predictions were taken from Guner et al. (2022) [33].

**Figure 8:**
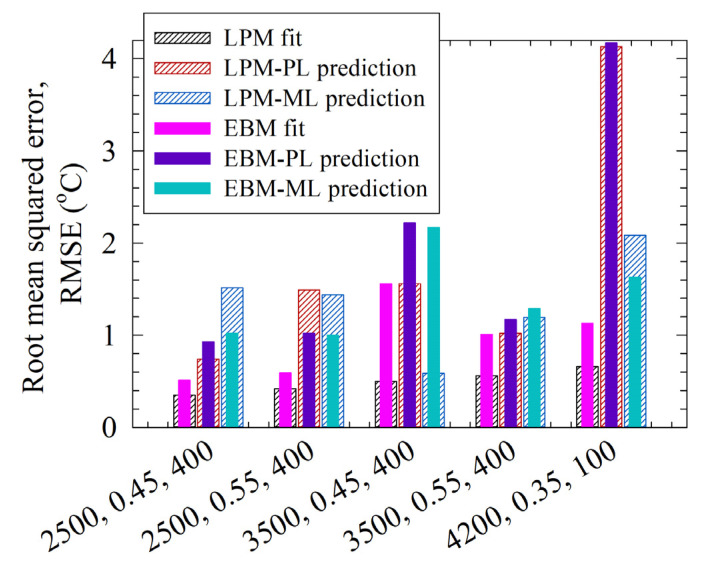
Comparison of the root-mean-squared errors of the direct fits and the predictions made by the power law (PL) and machine learning (ML) models coupled with the lumped parameter model (LPM) and the enthalpy balance model (EBM). The EBM predictions were taken from Guner et al. (2022) [33].

The LPM–PL and LPM–ML model predictions are compared with the EBM-PL and the EBM–ML predictions, which were retrieved from Guner et al. [33], as seen in Figure 7 and Figure 8. Whereas all LPM predictions approached an asymptote as the experimental data did, the EBM predictions tended to have a maximum temperature (Figure 7). Moreover, there are better and worse prediction examples when the LPM and EBM predictions are compared. The average ± standard deviation of the RMSEs are 1.57 ± 0.99 °C for all LPM predictions and 1.66 ± 1.00 °C for all EBM predictions. Overall, the LPM is better for predictions but based on the average RMSEs, the difference is not as drastic as it is when the RMSEs associated with direct fittings are compared.

The average ± standard deviation of the RMSEs are 1.79 ± 1.35 °C and 1.36 ± 0.54 °C, respectively, for the LPM–PL and LPM–ML predictions. The LPM–PL exhibited better or similar predictive capability for most of the test runs, as can be seen from Figure 8, which represents the comparative RMSEs visually. However, the LPM–PL prediction for Run 32 was notably bad. A similarly bad prediction was made by the EBM–PL for the same run. Interestingly, despite its larger deviations for the training runs, when augmented with either the LPM or the EBM, the prediction with the ML model had much lower RMSE than that with the PL model for Run 32. The process conditions of this run were purposefully chosen to be outside the domain of the training runs to test the models under extreme cases. They are not likely used in a lab or industrial setting due to unrealistically low bead loading and very small bead sizes. In general, small beads, e.g., 100 µm, are more difficult to handle compared with 300–600 µm beads during the operations. Nonetheless, these findings suggest that either the EBM or the LPM should be used with caution outside the domain of the training runs.

### 3.5. The LPM and the EBM Comparison and the Limitations of the LPM

The full EBM developed by Guner et al. [33] is a comprehensive model of the heat generation–transfer in WSMM that considers recirculation and batch size as well as the cooling rate provided by the jackets of the milling chamber and the holding tank. It entails using the values of power consumption, physico-chemical properties of the fluid and the beads, and the dimensions of the mill setup, which were needed for the calculation of the overall heat transfer coefficient *U* and heat transfer surface area of the mill chamber *A*_m_ (refer to [33] for details of the EBM). The EBM is so versatile that it can be used to investigate the impacts of different coolant types and flow rates as well as the material of construction of the mill on the cooling rate and temperature evolution. Despite all these capabilities and higher fidelity to the actual milling process, its use entails more time, effort, and accurate numerical methods for the simulations/parameter estimation. Moreover, one must obtain appropriate data and correlations for the physical–thermal–heat transfer properties. The EBM consists of five ordinary differential equations (ODEs) with the fraction *ξ* of the power consumption *P* that is converted into heat being the sole parameter of the EBM. The estimation of *ξ* entails using a sophisticated optimizer coupled to the ODE solver. Hence, for facile modeling of the temperature profiles as well as effective control and optimization of the WSMM process, development of simpler, low-fidelity models such as the LPM may be warranted.

In contrast to the EBM, the LPM is a simple semi-theoretical model, with two adjustable parameters, which was developed based on various assumptions mentioned in Section 2.2.2. Hence, it has a closed-form analytical solution and can be easily used without much effort and time, while obviating the need for sophisticated ODE solvers/optimizers. The main limitation of the LPM is that it is only valid for a given mill set-up with constant batch size and recirculation rate. This aspect may not be critical because, to the best knowledge of the authors, these parameters are usually kept invariant whereas the impacts of the stirrer speed, the bead loading, the bead size, and milling time have been investigated in most WSMM process studies (see e.g., the reviews [15,16]). Another limitation of the LPM is that although it can predict the temperature of the suspension in the milling chamber, it does not provide any information about the temperatures of the suspension in the holding tank, temperatures of the beads, the stirrer of the mill chamber, and the stirrer of the holding tank. Contrarily, the EBM predicted a lower temperature of the suspension in the holding tank than that in the milling chamber and established a thermal equilibrium among the suspension, the stirrer element, and the beads, owing to fast convective heat transfer and relatively small size of the beads and the mill stirrer [33].

Despite its simplicity, when augmented with the PL and the ML models, the LPM predictions of the temperature profiles in the test runs were better than or similar to the EBM predictions (refer to Figure 7 and Figure 8). Moreover, the LPM predicts a monotone increasing temperature profile with a steady-state temperature approach for sufficiently long milling, which is in excellent qualitative agreement with the profiles in Figure 3, Figure 4, Figure 5, Figure 6 and Figure 7. In fact, Equation (2) clearly shows that in the limit *t*→∞, the temperature reaches a steady-state temperature of *T*_ch_ + *Q*_gen_/*UA*. Although the EBM has higher fidelity to the real WSMM operation, its predictions could be worse, and some predictions exhibited a maximum and ensuing drop in temperature instead of a monotonic approach to a steady-state in the temperature profiles (Figure 7). In general, the temperature drop from the maximum is within a couple of degrees Celsius, and this error was acceptable. Note that even the EBM has its own assumptions and sources of modeling errors; refer to Guner et al. [33] for a detailed discussion of the modeling errors. For example, in the *UA*_m_ calculations, the mixture correlations for the physical properties and the internal/external convective heat transfer coefficients have some errors. The LPM is free of that source of modeling error because *UA* was used as a fitting parameter; therefore, having two fitting parameters, as opposed to the one fitting parameter of the EBM, enhanced LPM’s fitting and prediction capability.

The main drawback of the LPM is that it does not consider the enthalpic effects associated with the recirculation of the drug suspension and the thermal inertia effects associated with the batch volume of the suspension in the holding tank. Precisely because of this simplification, the LPM is referred to as a semi-theoretical model here, which considers some physical aspects of the process in some time-average, approximate, and statistical sense. Its *Q*_gen_ and *UA* are fitting parameters that are not equal to the true heat generation rate and the product of the overall heat transfer coefficient and the heat transfer surface area. In fact, the actual heat generation rate and even the overall heat transfer coefficient vary with time and temperature [33,34]. However, as established in this study, *Q*_gen_ is strongly and positively correlated with the power consumption and is the driver for temperature rise, whereas the *UA* correlation with the process parameters revealed a similar qualitative dependence of the convective heat transfer coefficients on the process parameters in their correlations (refer to such correlations in [33]).

The LPM’s treatment of the recirculation operation as an equivalent batch operation seems unreasonable for the modeling of temperature profiles on purely theoretical grounds. Being aware of this limitation, we accept the resulting modeling error for the sake of simplicity as LPM is intended to be a low-fidelity model for industrial use. It is also worth mentioning that a similar approach has already been adopted for the modeling of the evolution of the median particle size and even the whole particle size distribution during the recirculation operation of the WSMM by multiple research groups (e.g., [29,73,74]).

### 3.6. A holistic Perspective on the Impact of Process Parameters

This study has focused on the impact of process parameters on heat generation and temperature evolution during the WSMM and a comparative analysis of the newly-developed LPM and the recently developed EBM. In a previous study, Guner et al. [34] investigated the impact of the process parameters on the particle sizes of the milled suspensions (refer to Table S2). An increase in the stirrer speed and bead loading led to smaller *d*_50_ and *d*_90_, whereas an increase in the bead size led to bigger *d*_50_ and *d*_90_. As the increase in the stirrer speed and bead loading also leads to higher heat generation rate and temperature rise, existence of an optimal set of process conditions is anticipated. Clearly, the milling conditions that are conducive to higher milling efficiency also cause higher heat generation and temperature rise. This is not surprising as both of them are largely determined by the mechanical power consumption during milling. Guner et al. [34] considered a desirable product specification of *d*_10_ < 150 nm, *d*_50_ < 200 nm, and *d*_90_ < 250 nm and max. temperature below 37 °C. Their holistic consideration of a newly-defined thermal desirability score, power (energy) consumption, and total cycle time suggested that Run 13 or 14 had the optimal set of milling conditions: 3000 rpm with 50% loading of 200 or 400 µm beads. Obviously, in general, the optimal conditions depend on the specific pharmaceutical application of the drug nanosuspension with desired particle size specifications as well as the sensitivity of the drug to temperature and stressing in terms of physical stability and chemical degradation.

## 4. Conclusions and Future Outlook

This study has developed and implemented a semi-theoretical, lumped-parameter model (LPM) as an alternative to the recently developed enthalpy balance model (EBM) for simulating and predicting the temperature evolution during the nanomilling of drug suspensions. It has two fitting parameters which make it more flexible compared to the enthalpy balance model; therefore, it provides better fitting results. Although the apparent heat generation rate and the apparent heat transfer coefficient multiplied by the heat transfer surface area are not equal to the actual values, they could be predicted by process conditions via the machine learning (ML) and the power-law (PL) approaches. The fittings and predictions of the LPM were found to be slightly better than those of the EBM. Overall, our experimental and modeling results suggest that the LPM has excellent descriptive/fitting capability in addition to its reasonably good predictive capability. Coupled with its simplicity, which obviates the need for using a sophisticated coupled optimizer–ODE solver, it could be selected for facile modeling of the WSMM process. Hence, we provide the pharmaceutical engineering literature with two different models, the LPM and the EBM, which can be used on a fit-for-purpose basis. If a quick analysis and modeling of the temperature profiles are needed without significant effort/time, then the LPM is advantageous and should be used. The LPM can also be easily used for process control. However, if the aim is to intensify and optimize the process that entails a detailed and deep understanding of the impact of the recirculation rate, batch size, cooling type/capacity, and material of construction, then the EBM must be used. In that case, power consumption and the physico-chemical and thermal properties of the suspensions and the beads, as well as reliable correlations for the convective heat transfer coefficient, must be obtained from the literature and/or determined experimentally. Future work will involve applying the LPM and EBM to pharmaceutical wet media milling processes wherein (i) polymeric beads as opposed to ceramic beads are used, (ii) a large batch size (e.g., 7 L) of drug suspensions is to be milled, and (iii) pilot and large-scale commercial wet media mills are used.

## Figures and Tables

**Table 1:** Process parameters for the milling of FNB suspensions.

Run No.	Stirrer Speed, *ω* (rpm)	Bead Loading, *c* (-)	Bead Size, *D*_b_ (µm)
1 ^1^	2000	0.4	200
2 ^1^	2000	0.4	400
3 ^1^	2000	0.4	800
4 ^1^	2000	0.5	200
5 ^1^	2000	0.5	400
6 ^1^	2000	0.5	800
7 ^1^	2000	0.6	200
8 ^1^	2000	0.6	400
9 ^1^	2000	0.6	800
10 ^1^	3000	0.4	200
11 ^1^	3000	0.4	400
12 ^1^	3000	0.4	800
13 ^1^	3000	0.5	200
14 ^1^	3000	0.5	400
15 ^1^	3000	0.5	800
16 ^1^	3000	0.6	200
17 ^1^	3000	0.6	400
18 ^1^	3000	0.6	800
19 ^1^	4000	0.4	200
20 ^1^	4000	0.4	400
21 ^1^	4000	0.4	800
22 ^1^	4000	0.5	200
23 ^1^	4000	0.5	400
24 ^1^	4000	0.5	800
25 ^1^	4000	0.6	200
26 ^1^	4000	0.6	400
27 ^1^	4000	0.6	800
28 ^2^	2500	0.45	400
29 ^2^	2500	0.55	400
30 ^2^	3500	0.45	400
31 ^2^	3500	0.55	400
32 ^2^	4000	0.35	100

^1^ Runs that were used in the training set, ^2^ Runs that were used in the test set.

**Table 2:** Fitted parameters of the LPM and associated statistics for the training runs.

Run No: Identifier	*Q*_gen_ (J/min)	*UA* (J/min °C)	RMSE (°C)
1: 2000 0.4 200	755.2	47.79	0.40
2: 2000 0.4 400	1616	103.5	0.46
3: 2000 0.4 800	787.4	48.08	0.56
4: 2000 0.5 200	441.4	25.82	0.32
5: 2000 0.5 400	720.8	40.18	0.33
6: 2000 0.5 800	1296	72.88	0.39
7: 2000 0.6 200	1343	68.63	0.44
8: 2000 0.6 400	2625	131.9	0.15
9: 2000 0.6 800	1822	89.95	0.50
10: 3000 0.4 200	1402	53.92	0.55
11: 3000 0.4 400	2938	107.0	0.57
12: 3000 0.4 800	2798	101.0	0.66
13: 3000 0.5 200	1837	61.49	0.40
14: 3000 0.5 400	3243	107.3	0.72
15: 3000 0.5 800	3307	107.4	0.83
16: 3000 0.6 200	2624	94.08	0.43
17: 3000 0.6 400	4598	128.2	0.76
18: 3000 0.6 800	4542	124.4	0.80
19: 4000 0.4 200	5075	135.1	0.42
20: 4000 0.4 400	6266	162.7	0.41
21: 4000 0.4 800	6245	162.5	0.51
22: 4000 0.5 200	6116	162.7	0.90
23: 4000 0.5 400	8490	220.0	0.30
24: 4000 0.5 800	9359	238.1	0.60
25: 4000 0.6 200	8383	208.9	0.62
26: 4000 0.6 400	10,600	261.7	0.28
27: 4000 0.6 800	10,740	171.9	0.34

**Table 3:** Parameters of the LPM estimated by direct fitting as well as predicted using the PL and ML models coupled to the LPM along with the associated statistics.

Runs	Direct Fitting				PL Prediction				ML Prediction			
	*Q*_gen_ (J/min)	*UA* (J/min °C)	LPM RMSE (°C)	EBM RMSE (°C) ^1^	*Q*_gen_ (J/min)	*UA* (J/min °C)	LPM RMSE (°C)	EBM RMSE (°C) ^1^	*Q*_gen_ (J/min)	*UA* (J/min °C)	LPM RMSE (°C)	EBM RMSE (°C) ^1^
2500 0.45 400	1481	68	0.35	0.51	1634	78	0.74	0.93	1792	77	1.51	1.02
2500 0.55 400	2495	101	0.42	0.59	2118	91	1.49	1.02	2506	95	1.44	1.00
3500 0.45 400	4084	118	0.50	1.56	4519	137	1.56	2.22	4555	132	0.59	2.17
2500 0.55 400	5567	147	0.56	1.01	5856	160	1.02	1.17	5911	162	1.19	1.29
4200 0.35 100	3185	84	0.66	1.13	4180	129	4.13	4.17	4359	124	2.08	1.63

^1^ RMSE data were taken from Guner et al. 2022 [33] for comparison with the LPM.

## Data Availability

Data is contained within the article and supplementary material.

## Supplementary Materials

The following supporting information can be downloaded at: https://www.mdpi.com/article/10.3390/pharmaceutics14122840/s1, Table S1: Mean squared error (MSE) and mean absolute error (MAE) of the model predictions for *Q*_gen_ and *UA* in the training and test tests; Table S2: Particle size statistics for the milled suspensions.

## Abbreviations

*A*	heat transfer surface area, m^2^
*A*_m_	heat transfer surface area of the mill chamber, m^2^
*c*	bead loading or fractional volumetric concentration of the beads, –
*C*_p_	specific heat capacity, J/g °C
EBM	enthalpy balance model
*D*_b_	bead size, µm
LPM	lumped-parameter model
m	mass in the mill chamber, g
ML	machine learning
PL	power law
*P*	power consumption, J/min
*Pξ*	heat generation rate during milling, retrieved from EBM study, J/min
*Q*_gen_	apparent heat generation during milling, J/min
RMSE	root-mean-squared error, °C
*T*	temperature at the mill outlet, °C
*T*_ch_	chiller temperature, °C
*T*_rise_	temperature rise at 6 min of milling, °C
*T*_0_	initial temperature at the mill outlet, °C
*t*	milling time, min
*U*	overall heat transfer coefficient, W/m^2^ °C
*UA*	apparent overall heat transfer coefficient times surface area, J/min °C
*UA*_m_	apparent overall heat transfer coefficient times surface area in the mill chamber, J/min °C
*ξ*	fraction of mechanical power converted into heat, –
*ω*	stirrer (rotational) speed, rpm

## References

[1] Jermain S.V., Brough C., Williams R.O. (2018). Amorphous solid dispersions and nanocrystal technologies for poorly water-soluble drug delivery—An update. Int. J. Pharm..

[2] Ku M.S., Dulin W. (2012). A biopharmaceutical classification-based right-first-time formulation approach to reduce human pharmacokinetic variability and project cycle time from first-in-human to clinical proof-of-concept. Pharm. Dev. Technol..

[3] Takagi T., Ramachandran C., Bermejo M., Yamashita S., Yu L.X., Amidon G.L. (2006). A provisional biopharmaceutical classification of the top 200 oral drug products in the United States, great Britain, Spain, and Japan. Mol. Pharm..

[4] Kipp J. (2004). The role of solid nanoparticle technology in the parenteral delivery of poorly water-soluble drugs. Int. J. Pharm..

[5] Anane-Adjei A.B., Jacobs E., Nash S.C., Askin S., Soundararajan R., Kyobula M., Booth J., Campbell A. (2022). Amorphous solid dispersions: Utilization and challenges in preclinical drug development within astrazeneca. Int. J. Pharm..

[6] Keserü G.M., Makara G.M. (2009). The influence of lead discovery strategies on the properties of drug candidates. Nat. Rev. Drug Discov..

[7] Ricarte R.G., van Zee N.J., Li Z., Johnson L.M., Lodge T.P., Hillmyer M.A. (2019). Recent advances in understanding the micro- and nanoscale phenomena of amorphous solid dispersions. Mol. Pharm..

[8] Lipinski C.A., Lombardo F., Dominy B.W., Feeney P.J. (2012). Experimental and computational approaches to estimate solubility and permeability in drug discovery and development settings. Adv. Drug Deliv. Rev..

[9] Liu H., Taylor L.S., Edgar K.J. (2015). The role of polymers in oral bioavailability enhancement; a review. Polymer.

[10] Tan J., Liu J., Ran L. (2021). A review of pharmaceutical nano-cocrystals: A novel strategy to improve the chemical and physical properties for poorly soluble drugs. Crystals.

[11] Bhujbal S.V., Mitra B., Jain U., Gong Y., Agrawal A., Karki S., Taylor L.S., Kumar S., Zhou Q.T. (2021). Pharmaceutical amorphous solid dispersion: A review of manufacturing strategies. Acta Pharm. Sin. B.

[12] Schittny A., Huwyler J., Puchkov M. (2020). Mechanisms of increased bioavailability through amorphous solid dispersions: A review. Drug Deliv..

[13] Sathisaran I., Dalvi S.V. (2018). Engineering cocrystals of poorly water-soluble drugs to enhance dissolution in aqueous medium. Pharmaceutics.

[14] Kumar S. (2018). Pharmaceutical cocrystals: An overview. Indian J. Pharm. Sci..

[15] Bhakay A., Rahman M., Dave R.N., Bilgili E. (2018). Bioavailability enhancement of poorly water-soluble drugs via nanocomposites: Formulation–processing aspects and challenges. Pharmaceutics.

[16] Li M., Azad M., Davé R., Bilgili E. (2016). Nanomilling of drugs for bioavailability enhancement: A holistic formulation-process perspective. Pharmaceutics.

[17] Bilgili E., Guner G. (2020). Mechanistic modeling of wet stirred media milling for production of drug nanosuspensions. AAPS PharmSciTech.

[18] Lehocký R., Pěček D., Štěpánek F. (2016). Scale-up from batch to flow-through wet milling process for injectable depot formulation. Eur. J. Pharm. Sci..

[19] Nakach M., Authelin J.-R., Agut C. (2017). New approach and practical modelling of bead milling process for the manufacturing of nanocrystalline suspensions. J. Pharm. Sci..

[20] Siewert C., Moog R., Alex R., Kretzer P., Rothenhäusler B. (2018). Process and scaling parameters for wet media milling in early phase drug development: A knowledge based approach. Eur. J. Pharm. Sci..

[21] Breitung-Faes S., Kwade A. (2008). Nano particle production in high-power-density mills. Chem. Eng. Res. Des..

[22] Hennart S.L.A., Domingues M.C., Wildeboer W.J., van Hee P., Meesters G.M.H. (2010). Study of the process of stirred ball milling of poorly water soluble organic products using factorial design. Powder Technol..

[23] Juhnke M., Märtin D., John E. (2012). Generation of wear during the production of drug nanosuspensions by wet media milling. Eur. J. Pharm. Biopharm..

[24] Singare D.S., Marella S., Gowthamrajan K., Kulkarni G.T., Vooturi R., Rao P.S. (2010). Optimization of formulation and process variable of nanosuspension: An industrial perspective. Int. J. Pharm..

[25] Singh S.K., Srinivasan K., Gowthamarajan K., Singare D.S., Prakash D., Gaikwad N.B. (2011). Investigation of preparation parameters of nanosuspension by top-down media milling to improve the dissolution of poorly water-soluble glyburide. Eur. J. Pharm. Biopharm..

[26] Patel P.J., Gajera B.Y., Dave R.H. (2018). A quality-by-design study to develop nifedipine nanosuspension: Examining the relative impact of formulation variables, wet media milling process parameters and excipient variability on drug product quality attributes. Drug Dev. Ind. Pharm..

[27] Ahuja B.K., Jena S.K., Paidi S.K., Bagri S., Suresh S. (2015). Formulation, optimization and in vitro–in vivo evaluation of febuxostat nanosuspension. Int. J. Pharm..

[28] Medarević D., Djuriš J., Ibrić S., Mitrić M., Kachrimanis K. (2018). Optimization of formulation and process parameters for the production of carvedilol nanosuspension by wet media milling. Int. J. Pharm..

[29] Afolabi A., Akinlabi O., Bilgili E. (2014). Impact of process parameters on the breakage kinetics of poorly water-soluble drugs during wet stirred media milling: A microhydrodynamic view. Eur. J. Pharm. Sci..

[30] Guner G., Kannan M., Berrios M., Bilgili E. (2021). Use of bead mixtures as a novel process optimization approach to nanomilling of drug suspensions. Pharm. Res..

[31] Eskin D., Zhupanska O., Hamey R., Moudgil B., Scarlett B. (2005). Microhydrodynamics of stirred media milling. Powder Technol..

[32] Wylie J.J., Koch D.L., Ladd A.J. (2003). Rheology of suspensions with high particle inertia and moderate fluid inertia. J. Fluid Mech..

[33] Guner G., Elashri S., Mehaj M., Seetharaman N., Yao H.F., Clancy D.J., Bilgili E. (2022). An enthalpy-balance model for timewise evolution of temperature during wet stirred media milling of drug suspensions. Pharm. Res..

[34] Guner G., Seetharaman N., Elashri S., Mehaj M., Bilgili E. (2022). Analysis of heat generation during the production of drug nanosuspensions in a wet stirred media mill. Int. J. Pharm..

[35] Bitterlich A., Laabs C., Krautstrunk I., Dengler M., Juhnke M., Grandeury A., Bunjes H., Kwade A. (2015). Process parameter dependent growth phenomena of naproxen nanosuspension manufactured by wet media milling. Eur. J. Pharm. Biopharm..

[36] Knieke C., Azad M., Davé R., Bilgili E. (2013). A study of the physical stability of wet media-milled fenofibrate suspensions using dynamic equilibrium curves. Chem. Eng. Res. Des..

[37] Verma S., Kumar S., Gokhale R., Burgess D.J. (2011). Physical stability of nanosuspensions: Investigation of the role of stabilizers on ostwald ripening. Int. J. Pharm..

[38] Aleandri S., Schönenberger M., Niederquell A., Kuentz M. (2018). Temperature-induced surface effects on drug nanosuspensions. Pharm. Res..

[39] Descamps M., Willart J. (2016). Perspectives on the amorphisation/milling relationship in pharmaceutical materials. Adv. Drug Deliv. Rev..

[40] Coelho A., Schenck L., Guner G., Punia A., Bilgili E. (2022). A combined isolation and formulation approach to convert nanomilled suspensions into high drug-loaded composite particles that readily reconstitute. Powders.

[41] Khuman P., Singh W.B.K., Devi S.D., Naorem H. (2014). Viscosity-temperature behavior of hydroxypropyl cellulose solution in presence of an electrolyte or a surfactant: A convenient method to determine the cloud point of polymer solutions. J. Macromol. Sci. Part A.

[42] Frances C. (2004). On modelling of submicronic wet milling processes in bead mills. Powder Technol..

[43] Sommer M., Stenger F., Peukert W., Wagner N.J. (2006). Agglomeration and breakage of nanoparticles in stirred media mills—a comparison of different methods and models. Chem. Eng. Sci..

[44] Gudin D., Turczyn R., Mio H., Kano J., Saito F. (2006). Simulation of the movement of beads by the DEM with respect to the wet grinding process. AIChE J..

[45] Jayasundara C.T., Yang R.Y., Yu A.B. (2012). Discrete particle simulation of particle flow in a stirred mill: Effect of mill properties and geometry. Ind. Eng. Chem. Res..

[46] Varinot C., Berthiaux H., Dodds J. (1999). Prediction of the product size distribution in associations of stirred bead mills. Powder Technol..

[47] Stražišar J., Runovc F. (1996). Kinetics of comminution in micro- and sub-micrometer ranges. Int. J. Miner. Process..

[48] Azad M., Guner G., Afolabi A., Davé R., Bilgili E. (2021). Impact of solvents during wet stirred media milling of cross-linked biopolymer suspensions. Adv. Powder Technol..

[49] Guner G., Yilmaz D., Bilgili E. (2021). Kinetic and microhydrodynamic modeling of fenofibrate nanosuspension production in a wet stirred media mill. Pharmaceutics.

[50] Guner G., Yilmaz D., Eskin D., Bilgili E. (2022). Effects of bead packing limit concentration on microhydrodynamics-based prediction of breakage kinetics in wet stirred media milling. Powder Technol..

[51] Elsheikh A.H., Saba A.I., Panchal H., Shanmugan S., Alsaleh N.A., Ahmadein M. (2021). Artificial intelligence for forecasting the prevalence of covid-19 pandemic: An overview. Healthcare.

[52] Ahmadein M., Elsheikh A.H., Alsaleh N.A. (2022). Modeling of cooling and heat conduction in permanent mold casting process. Alex. Eng. J..

[53] Janssen A., Bennis F.C., Mathôt R.A. (2022). Adoption of machine learning in pharmacometrics: An overview of recent implementations and their considerations. Pharmaceutics.

[54] Jiang J., Ma X., Ouyang D., Williams III R.O. (2022). Emerging artificial intelligence (ai) technologies used in the development of solid dosage forms. Pharmaceutics.

[55] Jamzad S., Fassihi R. (2006). Role of surfactant and pH on dissolution properties of fenofibrate and glipizide—A technical note. AAPS PharmSciTech.

[56] Bilgili E., Rahman M., Palacios D., Arevalo F. (2018). Impact of polymers on the aggregation of wet-milled itraconazole particles and their dissolution from spray-dried nanocomposites. Adv. Powder Technol..

[57] NISSO Nisso HPC, Nippon Soda Co., Ltd. Nissoexcipients.Com. https://www.nissoexcipients.com/hpc-e/care_stable.

[58] Bilgili E., Li M., Afolabi A. (2016). Is the combination of cellulosic polymers and anionic surfactants a good strategy for ensuring physical stability of BCS class II drug nanosuspensions?. Pharm. Dev. Technol..

[59] Azad M., Afolabi A., Bhakay A., Leonardi J., Davé R., Bilgili E. (2015). Enhanced physical stabilization of fenofibrate nanosuspensions via wet co-milling with a superdisintegrant and an adsorbing polymer. Eur. J. Pharm. Biopharm..

[60] Li M., Yaragudi N., Afolabi A., Dave R., Bilgili E. (2015). Sub-100 nm drug particle suspensions prepared via wet milling with low bead contamination through novel process intensification. Chem. Eng. Sci..

[61] Li M., Alvarez P., Bilgili E. (2017). A microhydrodynamic rationale for selection of bead size in preparation of drug nanosuspensions via wet stirred media milling. Int. J. Pharm..

[62] Guner G. (2022). Microhydrodynamic, Kinetic and Thermal Modeling of Wet Media Milling for Process Optimization and Intensification. Ph.D. Dissertation.

[63] Geankoplis C. (2003). Transport Processes and Separation Process Principles.

[64] Annapragada A., Adjei A. (1996). Numerical simulation of milling processes as an aid to process design. Int. J. Pharm..

[65] Tojo T., Atake T., Mori T., Yamamura H. (1999). Heat capacity and thermodynamic functions of zirconia and yttria-stabilized zirconia. J. Chem. Thermodyn..

[66] James G., Witten D., Hastie T., Tibshirani R. (2013). An Introduction to Statistical Learning.

[67] Bilgili E., Afolabi A. (2012). A combined microhydrodynamics–polymer adsorption analysis for elucidation of the roles of stabilizers in wet stirred media milling. Int. J. Pharm..

[68] Cerdeira A.M., Gander B., Mazzotti M. (2011). Role of milling parameters and particle stabilization on nanogrinding of drug substances of similar mechanical properties. Chem. Eng. Technol..

[69] Fogel’Son R., Likhachev E. (2001). Temperature dependence of viscosity. Tech. Phys..

[70] Mannheim V. (2011). Empirical and scale-up modeling in stirred ball mills. Chem. Eng. Res. Des..

[71] Barrios A.N.S., Silva J.B.C., Rodrigues A.R., Coelho R.T., Braghini Junior A., Matsumoto H. (2014). Modeling heat transfer in die milling. Appl. Therm. Eng..

[72] Xi S.Q., Zhou J.G., Wang X.T. (2007). Research on temperature rise of powder during high energy ball milling. Powder Metall..

[73] Hennart S., Wildeboer W., van Hee P., Meesters G. (2009). Identification of the grinding mechanisms and their origin in a stirred ball mill using population balances. Chem. Eng. Sci..

[74] Li H., Ndjamo A., Sauriol P., Patience G.S. (2017). Optimization of lifepo4 wet media milling and regressive population balance modeling. Adv. Powder Technol..

