# Supplementary material for: Predicting the Temperature Evolution during Nanomilling of Drug Suspensions via a Semi-Theoretical Lumped-Parameter Model
Source: Pharmaceutics. 2022 Dec 18;14(12):2840. doi: 10.3390/pharmaceutics14122840 (PMC9788500; doi:10.3390/pharmaceutics14122840)
Supplement: Supplementary file 1 [file pharmaceutics-14-02840-s001.zip › pharmaceutics-2075515-supplementary.pdf]

Supplementary Material

Predicting the Temperature Evolution during Nanomilling of Drug Suspensions via a Semi-Theoretical Lumped-Parameter Model

Gulenay Guner [1], Dogacan Yilmaz [2], Helen F. Yao [3], Donald J. Clancy [3] and Ecevit Bilgili [1,*]

[1] Otto H. York Department of Chemical and Materials Engineering New Jersey Institute of Technology, Newark, NJ 07102, USA
[2] Department of Mechanical and Industrial Engineering, New Jersey Institute of Technology, Newark, NJ 07114, USA
[3] GlaxoSmithKline, Drug Product Development, Collegeville, PA 19426, USA
* Correspondence: bilgece@njit.edu

Table S1. Mean squared error (MSE) and mean absolute error (MAE) of the model predictions for Q_{gen} and *UA* in the training and test tests.

Model name	Q_{gen} (J/min)				*UA* (J/min°C)			
	Train		Test		Train		Test	
	MSE	MAE	MSE	MAE	MSE	MAE	MSE	MAE
Power law	3.28×10^5	4.64×10^2	2.86×10^5	4.50×10^2	8.28×10^2	22.0	5.48×10^2	19.4
Linear regression	1.27×10^6	9.23×10^2	1.55×10^6	9.27×10^2	9.53×10^2	24.0	1.00×10^3	22.2
Lasso regression	1.27×10^6	9.22×10^2	1.56×10^6	9.28×10^2	9.70×10^2	24.0	1.13×10^3	23.3
Ridge regression	1.55×10^6	9.98×10^2	1.27×10^6	8.86×10^2	1.05×10^3	25.0	8.52×10^2	19.9
Elastic net regression	6.00×10^6	2.03×10^3	1.66×10^6	1.15×10^3	2.57×10^3	40.1	8.44×10^2	24.8
Decision tree	0.00	0.00	2.69×10^6	1.45×10^3	0.00	0.00	1.84×10^3	39.4
Gradient boost	8.66×10^3	7.40×10^1	2.51×10^6	1.38×10^3	4.64×10^1	5.21	1.34×10^3	33.7
Random forest	1.18×10^5	2.72×10^2	3.01×10^6	1.50×10^3	1.60×10^2	10.0	1.63×10^3	36.3
K nearest neighborhood	1.58×10^6	9.30×10^2	3.63×10^5	4.62×10^2	8.88×10^2	22.4	4.32×10^2	16.9
Support vector regressor	1.09×10^7	2.48×10^3	2.11×10^6	1.18×10^3	3.66×10^3	46.3	6.83×10^2	22.4
Multilayer perceptron	2.37×10^7	3.79×10^3	1.01×10^7	3.11×10^3	2.07×10^3	39.5	1.06×10^3	29.4

Table S2. Particle size statistics for the milled suspensions.

Run no.	d_{10} (μm)	d_{50} (μm)	d_{90} (μm)
1	0.178 ± 0.003	0.314 ± 0.004	0.566 ± 0.014
2	0.190 ± 0.012	0.326 ± 0.011	0.592 ± 0.025
3	0.212 ± 0.008	0.400 ± 0.015	1.63 ± 0.016
4	0.158 ± 0.049	0.263 ± 0.009	0.454 ± 0.015
5	0.140 ± 0.006	0.296 ± 0.053	0.490 ± 0.015
6	0.185 ± 0.002	0.335 ± 0.007	0.661 ± 0.016
7	0.120 ± 0.001	0.204 ± 0.001	0.344 ± 0.002
8	0.123 ± 0.001	0.238 ± 0.002	0.382 ± 0.001
9	0.100 ± 0.011	0.241 ± 0.003	0.468 ± 0.011
10	0.122 ± 0.001	0.199 ± 0.006	0.325 ± 0.025
11	0.113 ± 0.002	0.221 ± 0.023	0.421 ± 0.016
12	0.167 ± 0.014	0.317 ± 0.019	0.642 ± 0.041
13	0.120 ± 0.005	0.174 ± 0.001	0.249 ± 0.001
14	0.121 ± 0.001	0.175 ± 0.001	0.246 ± 0.000
15	0.071 ± 0.002	0.223 ± 0.006	0.446 ± 0.015
16	0.111 ± 0.001	0.162 ± 0.001	0.235 ± 0.000
17	0.112 ± 0.001	0.162 ± 0.000	0.234 ± 0.001
18	0.102 ± 0.008	0.165 ± 0.005	0.258 ± 0.001
19	0.116 ± 0.004	0.172 ± 0.002	0.250 ± 0.001
20	0.111 ± 0.003	0.173 ± 0.002	0.256 ± 0.000
21	0.088 ± 0.005	0.244 ± 0.002	0.490 ± 0.003
22	0.109 ± 0.001	0.159 ± 0.001	0.232 ± 0.001
23	0.111 ± 0.001	0.162 ± 0.000	0.235 ± 0.000
24	0.105 ± 0.001	0.196 ± 0.001	0.384 ± 0.007
25	0.103 ± 0.002	0.149 ± 0.001	0.223 ± 0.001
26	0.106 ± 0.004	0.154 ± 0.003	0.229 ± 0.001
27	0.106 ± 0.007	0.179 ± 0.008	0.329 ± 0.054

[a]The standard deviation refers to multiple measurements by laser diffraction (n = 4). It is not a descriptor of a Gaussian particle size distribution. Adapted From Guner et al. (2022) [34].
